# Preoperative Neutrophil-to-Lymphocyte Ratio Is Associated with Lymphovascular Space Invasion and Nodal Metastasis in Vulvar Squamous Cell Carcinoma

**DOI:** 10.3390/cancers18162575

**Published:** 2026-08-11

**Authors:** Csongor Kárpáti, Richárd Tóth, Barbara Sebők, Pál Sebok, Attila Keszthelyi, Petra Merkely, Balázs Lintner, Lotti Lőczi, Nándor Ács, Márton Keszthelyi, Balázs Vida

**Affiliations:** 1 Department of Obstetrics and Gynecology, Semmelweis University, 1082 Budapest, Hungary; karpati.csongor@stud.semmelweis.hu (C.K.); toth.richard@semmelweis.hu (R.T.); sebok.pal.szabolcs@semmelweis.hu (P.S.); merkely.petra@semmelweis.hu (P.M.); lintner.balazs.zoltan@semmelweis.hu (B.L.); keszthelyi.lotti.lucia@semmelweis.hu (L.L.); acs.nandor@semmelweis.hu (N.Á.); vida.balazs.lajos@semmelweis.hu (B.V.); 2 Workgroup of Research Management, Doctoral School, Semmelweis University, 1085 Budapest, Hungary; sebok.barbara@semmelweis.hu; 3 Department of Urology, Semmelweis University, 1082 Budapest, Hungary; keszthelyi.attila@semmelweis.hu

**Keywords:** vulvar squamous cell carcinoma, neutrophil-to-lymphocyte ratio, lymph node metastasis, lymphovascular space invasion, inflammatory markers, clinicopathological associations

## Abstract

**Background:** Prognosis in vulvar squamous cell carcinoma (VSCC) is strongly influenced by lymph node involvement and adverse clinicopathological features. Systemic inflammatory indices derived from blood counts may reflect tumor–host interactions associated with adverse pathological phenotypes. **Objective:** To assess associations of preoperative neutrophil-to-lymphocyte ratio (NLR), platelet-to-lymphocyte ratio (PLR), and monocyte-to-lymphocyte ratio (MLR) with nodal metastasis, lymphovascular space invasion (LVSI), and tumor size. **Methods:** This retrospective single-center study included 93 surgically treated patients between March 2017 and February 2026. **Results:** NLR was associated with nodal metastasis (unadjusted *p* = 0.015; false discovery rate [FDR]-adjusted q = 0.047) and LVSI (unadjusted *p* < 0.001; FDR-adjusted q = 0.006). NLR showed modest discrimination for nodal metastasis (area under the receiver operating characteristic curve [AUC] = 0.647). For LVSI, NLR had the highest AUC among the evaluated markers (AUC = 0.789; 95% confidence interval [CI]: 0.683–0.894), but 16 cases were positive and the positive predictive value (PPV) was low (0.378). Nominal MLR findings for LVSI and large tumor size did not remain significant after FDR correction (both q = 0.308), while PLR showed no consistent associations. Elevated NLR was associated with nodal metastasis in univariable analysis (odds ratio [OR] = 3.291; *p* = 0.012) but not in multivariable models including LVSI (adjusted OR = 1.296; *p* = 0.654) or excluding LVSI (adjusted OR = 1.823; *p* = 0.261). **Conclusions:** NLR was associated with adverse pathological features but was not independently associated with nodal metastasis and should not be used as a standalone predictive or clinical decision-making tool.

## Simple Summary

Vulvar squamous cell carcinoma is a rare gynecological malignancy in which lymph node involvement is one of the most important prognostic factors. This study evaluated whether simple blood count-derived inflammatory markers, particularly the neutrophil-to-lymphocyte ratio (NLR), monocyte-to-lymphocyte ratio (MLR), and platelet-to-lymphocyte ratio (PLR), may help identify patients with adverse pathological features in vulvar cancer. We found that higher preoperative NLR values were associated with lymph node metastasis and lymphovascular space invasion (LVSI). However, elevated NLR was not independently associated with lymph node metastasis after adjustment for established clinicopathological factors. Among the evaluated markers, NLR had the highest AUC for LVSI, although the PPV was low. Nominal findings involving MLR did not remain significant after correction for multiple testing, while PLR showed no consistent associations. These findings suggest that NLR is an inexpensive and readily available inflammatory marker associated with an adverse pathological profile; however, it should not be considered a standalone predictor or replace established diagnostic and pathological evaluation methods.

## 1. Introduction

Patient outcomes in vulvar squamous cell carcinoma (VSCC) are strongly influenced by disease extent and nodal involvement. Reported 5-year disease-specific survival is substantially higher among patients without inguinofemoral lymph node metastasis, approximately 70–93%, whereas survival estimates decrease to about 25–41% in node-positive disease [1,2]. Accurate assessment of inguinofemoral lymph node status is therefore a central component of VSCC management. In selected patients with early stage VSCC, sentinel lymph node biopsy is recommended as a less morbid alternative to complete inguinofemoral lymphadenectomy. Current recommendations support its use in patients with unifocal International Federation of Gynecology and Obstetrics (FIGO) stage IB–II tumors < 40 mm, stromal invasion > 1 mm, and clinically negative groins. Sentinel lymph node mapping is commonly performed using technetium-99m combined with blue dye, while indocyanine green has also been increasingly used as a fluorescent tracer in vulvar cancer surgery [3,4,5]. Beyond its surgical relevance, nodal metastasis also reflects the biological behavior of the tumor, including its capacity for lymphovascular dissemination and interaction with the host immune-inflammatory response. In this context, preoperative blood count-derived inflammatory markers may reflect systemic inflammatory responses associated with nodal involvement in VSCC.

Systemic inflammatory biomarkers have emerged as promising candidates for prognostic assessment in several malignancies, as they reflect the interaction between tumor biology and the host immune response. Tumor-associated inflammation may promote cancer progression through multiple mechanisms, including enhancement of tumor growth, angiogenesis, invasion, and metastatic dissemination, while also inducing measurable alterations in circulating immune cell populations [6,7]. Consequently, inflammation-based biomarkers derived from peripheral blood have been widely investigated as prognostic indicators across various malignancies [6,8]. For example, recent studies in human papillomavirus (HPV)-associated cervical cancer and cervical intraepithelial lesions suggest that preoperative blood count-derived inflammatory markers may be associated with malignant conization outcomes and increasing histopathological severity [9,10,11,12,13]. However, data specifically addressing these markers in VSCC remain limited. Among these markers, the neutrophil-to-lymphocyte ratio (NLR) has attracted particular attention because it is one of the most extensively studied blood count-derived inflammatory indices and reflects the balance between neutrophil-driven inflammatory activation and lymphocyte-mediated antitumor immune activity. A higher NLR may therefore indicate a systemic shift toward innate inflammatory predominance, accompanied by a relatively weakened lymphocyte-mediated antitumor response. Accordingly, an increased NLR has been consistently associated with poorer survival outcomes in multiple solid tumors [8,14,15]. Importantly, its low cost and ease of assessment make it especially valuable in clinical settings with limited resources, where access to advanced diagnostic infrastructure may be restricted.

While NLR is among the most widely investigated complete blood count-derived inflammatory markers, complementary indices such as the monocyte-to-lymphocyte ratio (MLR) and the platelet-to-lymphocyte ratio (PLR) may further refine the evaluation of systemic inflammation and immune-related tumor responses. PLR reflects the interaction between platelet-mediated inflammatory activity and lymphocyte-mediated antitumor immunity, and elevated PLR has been associated with poorer outcomes in several gynecologic malignancies, including cervical, ovarian, and endometrial cancers [16,17,18,19,20]. MLR may similarly capture the balance between monocyte-related tumor-promoting inflammation and lymphocyte-dependent immune surveillance and has been reported as a prognostic marker in cervical and ovarian cancers [21,22,23,24].

Although NLR, PLR, and MLR have been investigated in several gynecologic malignancies, their relevance in vulvar squamous cell carcinoma remains insufficiently explored. Available evidence suggests that blood count-derived inflammatory markers may be associated with adverse clinicopathological features and advanced disease status in VSCC. However, the comparative value of NLR, PLR, and MLR in relation to nodal involvement, lymphovascular space invasion, and tumor size remains unclear. Therefore, the present study aimed to evaluate whether preoperative NLR, PLR, and MLR are associated with adverse clinicopathological characteristics in patients with VSCC.

## 2. Objectives

The primary objective of this study was to evaluate the association between preoperative blood count-derived inflammatory markers and lymph node metastasis in patients with vulvar squamous cell carcinoma. Secondarily, we investigated whether the NLR, PLR, and MLR were associated with lymphovascular space invasion and tumor size.

## 3. Materials and Methods

### 3.1. Patients

This single-institution retrospective cohort study included patients with histologically confirmed vulvar squamous cell carcinoma who were treated at the Department of Obstetrics and Gynecology, Semmelweis University, between March 2017 and February 2026. Cases were identified from institutional medical records and screened according to predefined clinical and laboratory eligibility criteria.

**Figure 1:**
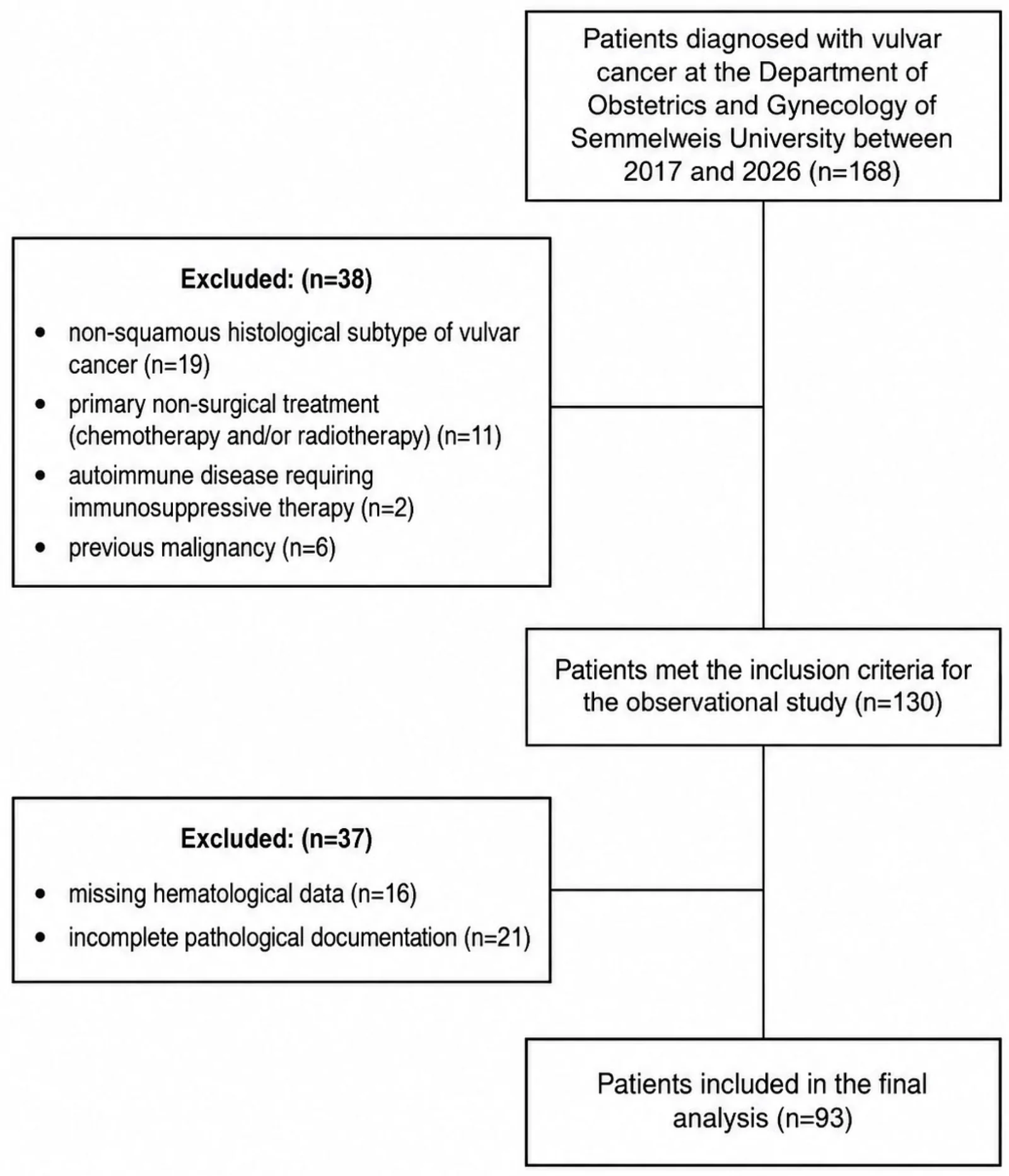
Flowchart of the patient selection. The diagram shows the stepwise inclusion and exclusion process of patients diagnosed with vulvar cancer between March 2017 and February 2026. After applying predefined exclusion criteria and removing cases with unavailable preoperative hematological data or incomplete pathological documentation, 93 patients were included in the final analysis.

A total of 168 patients with vulvar cancer were initially identified. Patients were eligible for inclusion if they had histologically confirmed invasive vulvar squamous cell carcinoma during the current diagnostic and treatment episode, available preoperative complete blood count results within one month before surgery, and sufficient clinical and pathological documentation for analysis. Histological eligibility was assessed using the complete pathology record, including both the initial diagnostic specimen and the definitive surgical specimen. Patients with invasive squamous cell carcinoma confirmed in the initial diagnostic specimen were considered to meet the inclusion criterion even when no residual invasive carcinoma was identified in the subsequent definitive surgical specimen. Exclusion criteria comprised autoimmune disease, ongoing immunosuppressive treatment, previous malignancy, prior surgery for vulvar malignancy, missing hematological data, incomplete pathological documentation, primary non-surgical treatment or a non-squamous histological subtype of vulvar cancer. Of the 168 patients initially screened, 75 were excluded according to mutually exclusive primary reasons for exclusion. Nineteen patients had a tumor diagnosis other than vulvar squamous cell carcinoma, including eight cases of extramammary Paget disease, four melanomas, and seven metastases or other carcinomas. Eleven patients received radiotherapy and/or chemotherapy and therefore did not meet the eligibility criteria for the surgically treated cohort. Two patients had autoimmune disease requiring immunosuppressive therapy, and six patients had a history of previous malignancy. In sixteen patients, the required preoperative hematological laboratory results were unavailable. A further twenty-one patients were excluded because the pathological documentation required for reliable ascertainment of the study variables was incomplete. After applying these criteria, 93 patients were included in the final analytical cohort. The patient selection process is summarized in Figure 1.

### 3.2. Characteristics

Demographic and baseline clinical variables included age and body mass index (BMI). BMI was calculated from preoperative height and weight measurements. Additional clinical variables included relevant comorbidities, previous vulvar disease, and gynecological history, when available. Pathological variables were extracted from the complete pathology documentation of the current diagnostic and treatment episode, including both the initial diagnostic specimen and the definitive surgical specimen, and included tumor grade, FIGO stage, tumor size, depth of stromal invasion, lymphovascular space invasion, surgical margin status, and lymph node involvement. For consistency, FIGO stage was assigned according to the 2021 FIGO staging system for all patients, including the retrospective restaging of cases treated before 2021 based on the available clinical and pathological documentation. In patients with histologically confirmed invasive squamous cell carcinoma in the initial diagnostic specimen but no residual invasive carcinoma in the definitive surgical specimen, the complete pathology record was used for histological classification and eligibility assessment. Tumor size was defined as the largest recorded linear dimension of the invasive tumor. Nodal status was classified according to the presence or absence of histologically confirmed lymph node metastasis. These parameters were selected because of their established relevance for disease extent and prognosis in vulvar squamous cell carcinoma.

Preoperative hematological parameters were obtained from complete blood count results recorded before surgical treatment. When multiple preoperative measurements were available, the value closest to the date of surgery was used, provided that it had been obtained within one month before the procedure. Absolute neutrophil and lymphocyte counts were recorded, and the neutrophil-to-lymphocyte ratio was calculated by dividing the absolute neutrophil count by the absolute lymphocyte count. Platelet-to-lymphocyte ratio was calculated by dividing the absolute platelet count by the absolute lymphocyte count, and monocyte-to-lymphocyte ratio was calculated by dividing the absolute monocyte count by the absolute lymphocyte count. Absolute neutrophil, lymphocyte, and monocyte counts are reported in G/L, whereas platelet counts are reported in T/L. For PLR calculation, platelet counts were converted to G/L before division by the lymphocyte count. NLR, PLR, and MLR are therefore dimensionless ratios.

### 3.3. Data Management

Clinical, pathological, and laboratory data were retrospectively extracted from patient records and entered into a study specific database. The dataset was reviewed before analysis to identify missing values, inconsistencies, and potential recording errors. Incomplete data were managed according to predefined criteria to support reliable statistical evaluation. Follow-up time was recorded as documented in the medical records, while calculated follow-up time was derived retrospectively from the date of surgery to the last available follow-up or death, which explains minor differences between the two variables.

### 3.4. Statistical Analysis

**Figure 2:**
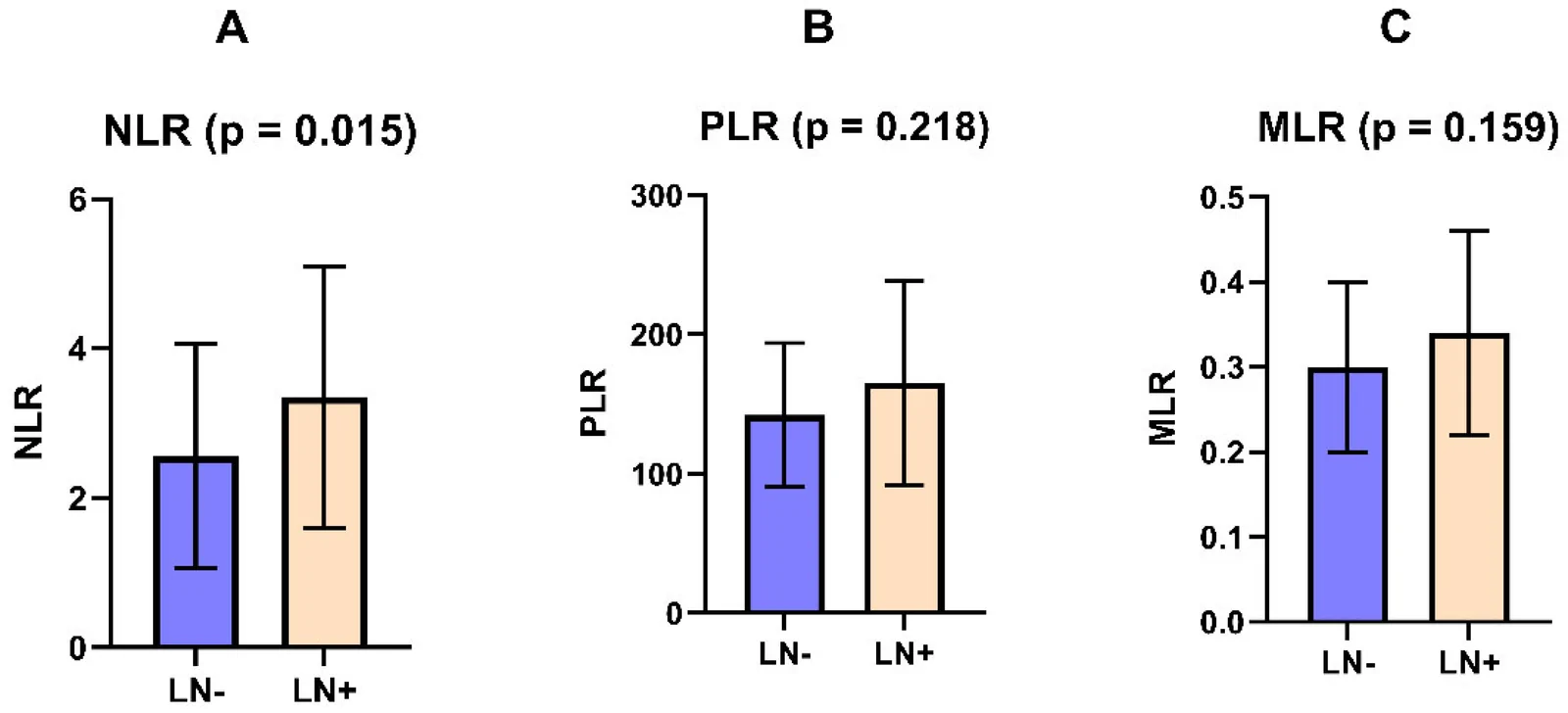
Preoperative inflammatory markers according to lymph node metastasis status. (**A**) NLR values in patients without (LN−) and with (LN+) lymph node metastasis; (**B**) PLR values in patients without (LN−) and with (LN+) lymph node metastasis; (**C**) MLR values in patients without (LN−) and with (LN+) lymph node metastasis. Values are presented as mean ± standard deviation. Neutrophil, lymphocyte, and monocyte counts were expressed in G/L, whereas platelet counts were expressed in T/L. For PLR calculation, platelet counts were converted to G/L before division by the lymphocyte count. NLR, PLR, and MLR are dimensionless ratios. Displayed *p* values are unadjusted and were calculated using the Mann–Whitney U test; false discovery rate (FDR)-adjusted results are reported in the text.

**Figure 3:**
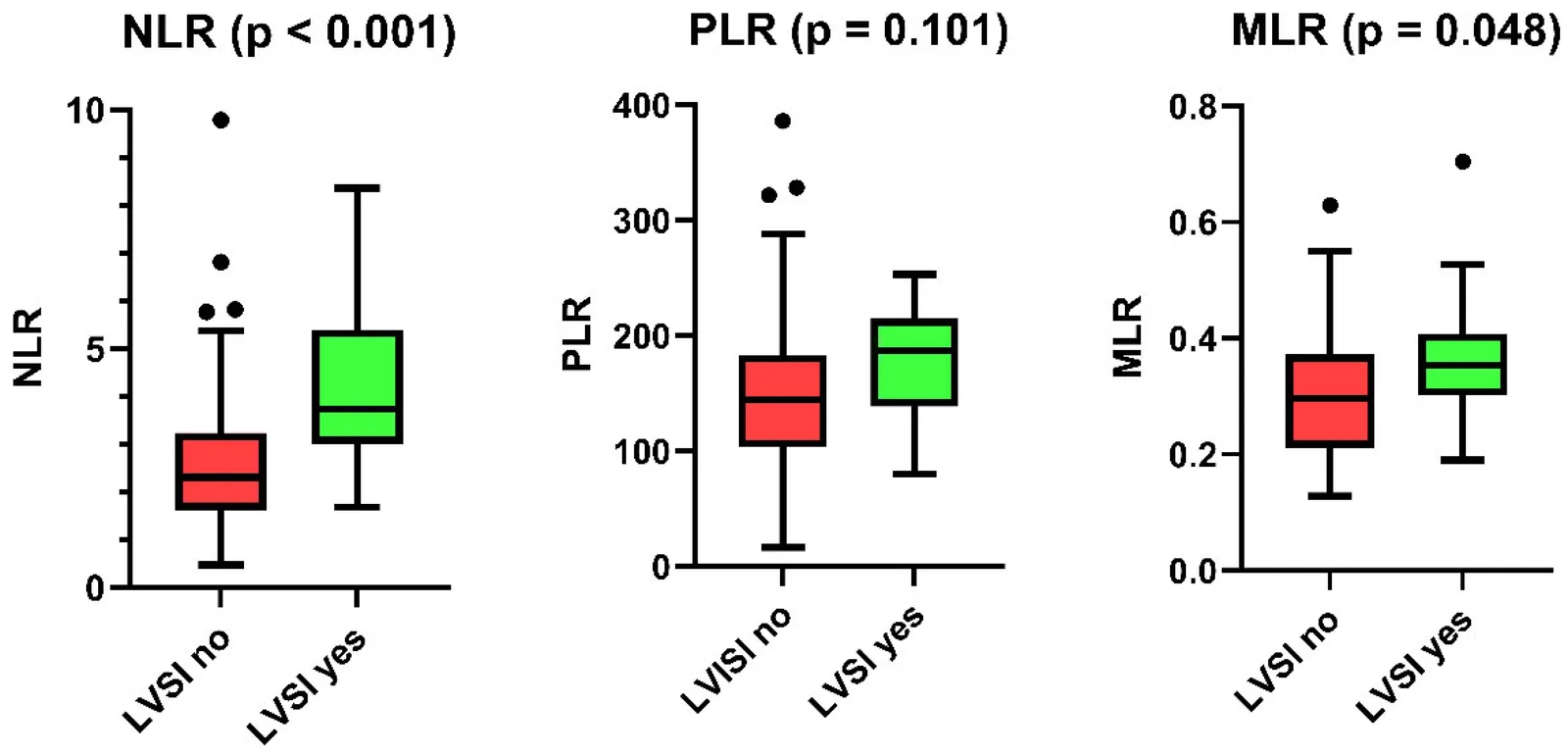
Preoperative inflammatory markers according to LVSI status. Tukey boxplots show the distribution of calculated NLR, PLR, and MLR values in patients with and without lymphovascular space invasion. Neutrophil, lymphocyte, and monocyte counts were expressed in G/L, whereas platelet counts were expressed in T/L. For PLR calculation, platelet counts were converted to G/L before division by the lymphocyte count. NLR, PLR, and MLR are dimensionless ratios. NLR remained significantly associated with LVSI after FDR correction, whereas MLR showed only a nominal unadjusted association and PLR was not significant. Displayed *p* values are unadjusted and were calculated using the Mann–Whitney U test; FDR-adjusted results are reported in the text.

**Figure 4:**
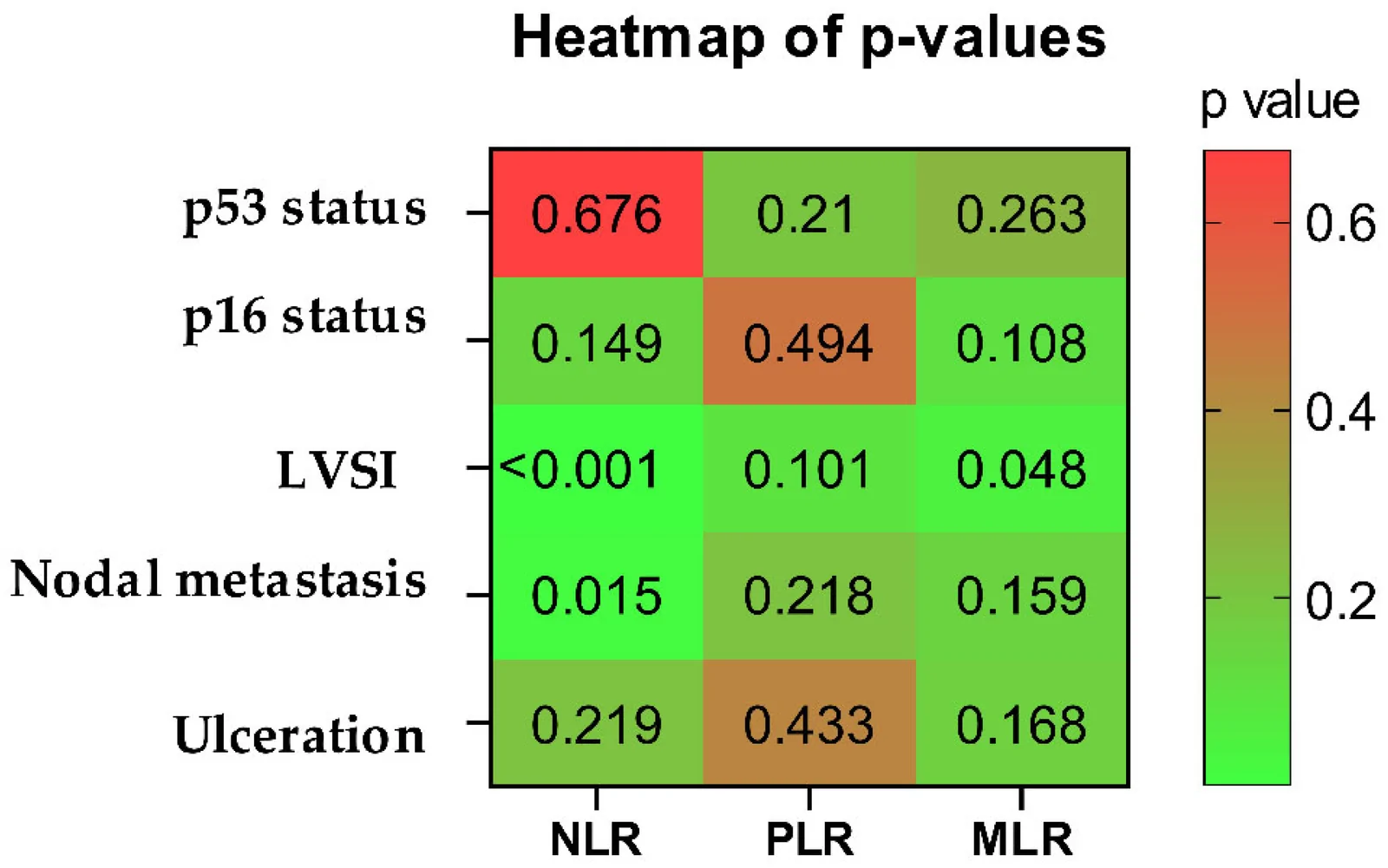
Heatmap of unadjusted *p* values for associations between inflammatory markers and binary clinicopathological variables. The heatmap summarizes unadjusted *p* values for the associations between NLR, PLR, and MLR and selected binary clinicopathological variables. Lower values indicate stronger statistical associations. The heatmap is descriptive and exploratory; FDR-adjusted results are reported in the text. The associations between inflammatory markers and continuous clinicopathological variables were assessed using Spearman’s rank correlation analysis. NLR showed a weak positive but statistically non-significant correlation with largest tumor dimension (ρ = 0.190, *p* = 0.073), indicating that higher NLR values were not significantly associated with larger tumor size. Similarly, PLR (ρ = 0.114, *p* = 0.286) and MLR (ρ = 0.133, *p* = 0.215) were not significantly correlated with largest tumor dimension. None of the evaluated inflammatory markers showed a significant correlation with maximum depth of stromal invasion. The detailed correlation results are presented in Table 3.

All statistical evaluations were carried out in IBM SPSS Statistics for Windows, Version 25.0 (Released 2017, IBM Corp., Armonk, NY, USA). Figures 2–4 were prepared using GraphPad Prism, Version 11.0.2 (GraphPad Software, Boston, MA, USA). Continuous variables were summarized using mean, standard deviation, median, and interquartile range (IQR), as appropriate. Categorical variables were presented as frequencies and percentages. The normality of continuous variables was assessed using the Shapiro–Wilk test. As most hematological and clinicopathological variables showed non-normal distribution, non-parametric statistical methods were applied. Preoperative inflammatory markers, including the neutrophil-to-lymphocyte ratio (NLR), platelet-to-lymphocyte ratio (PLR), and monocyte-to-lymphocyte ratio (MLR), were evaluated in relation to clinicopathological parameters. Differences in inflammatory marker values according to binary clinicopathological variables, including lymphatic metastasis and lymphovascular space invasion (LVSI), were assessed using the Mann–Whitney U test. Correlations between inflammatory markers and continuous clinicopathological variables, including largest tumor dimension and maximum depth of stromal invasion, were analyzed using Spearman’s rank correlation coefficient. To evaluate the discriminatory performance of preoperative inflammatory markers for adverse clinicopathological outcomes, receiver operating characteristic (ROC) curve analyses were performed, and the area under the curve (AUC) with 95% confidence interval (CI) was calculated. ROC analysis was first performed to evaluate the discriminatory performance of NLR for lymphatic metastasis, which represented the primary outcome of the study. An exploratory subgroup ROC analysis was additionally performed in patients with FIGO stage IB–II disease and evaluable nodal status. Additional ROC analyses were performed to assess the discriminatory performance of NLR, PLR, and MLR for the presence of lymphovascular space invasion (LVSI) and large tumor size, defined as the largest tumor dimension > 40 mm. For lymphatic metastasis, optimal NLR cut-off values were determined using both the Youden index and the Closest Top-Left method. For LVSI and large tumor size, Youden index-based cut-off values were reported. All cut-off values were derived within the study cohort and were not internally or externally validated. The corresponding diagnostic performance measures therefore represent apparent in-sample estimates. For the identified cut-off values, sensitivity, 1-specificity, positive predictive value (PPV), negative predictive value (NPV), positive likelihood ratio (+LR), negative likelihood ratio (−LR), and overall accuracy were calculated. Patients were subsequently categorized according to the Youden-based NLR cut-off value. The association between elevated NLR and lymphatic metastasis was assessed using cross-tabulation analysis, Pearson’s chi-square test, and Fisher’s exact test, where appropriate. Odds ratios (ORs) with 95% CIs were calculated to estimate the strength of association. Binary logistic regression analysis was performed to further evaluate the association between elevated NLR and lymphatic metastasis. Univariable logistic regression was first used to assess the crude association between NLR ≥ 3.1 and lymphatic metastasis. Two multivariable logistic regression models were constructed to evaluate the association between elevated NLR and lymph node metastasis after adjustment for clinically relevant clinicopathological factors. Both models included elevated NLR, largest tumor dimension, tumor grade, and maximum depth of stromal invasion. Model 1 additionally included LVSI, whereas Model 2 excluded LVSI because of its close biological relationship with nodal dissemination and its potential role as an intermediate pathological variable. The multivariable models were intentionally restricted to a limited number of clinically relevant variables because of the relatively small sample size and event count, in order to reduce the risk of model overfitting and unstable parameter estimates. Model performance and fit were assessed using the omnibus test, Cox–Snell R^2^, Nagelkerke R^2^, the Hosmer–Lemeshow goodness-of-fit test, and classification accuracy. To account for multiple marker–outcome comparisons, the Benjamini–Hochberg procedure was applied separately to the three marker comparisons for the primary endpoint and to the remaining 21 exploratory comparisons. A false discovery rate (FDR)-adjusted q value < 0.05 was considered statistically significant. Missing data were handled by complete-case analysis for each statistical procedure. All tests were two-sided, and *p* values < 0.05 were considered statistically significant.

## 4. Results

The baseline characteristics of the included patients are summarized in Table 1. In total, 93 patients were included in the final analysis. The mean age was 68.43 ± 11.22 years (median: 70), and the mean body mass index (BMI) was 28.14 ± 5.46 kg/m^2^ (median: 27.85). Hypertension was present in 68.8% of patients, while 22.6% had diabetes mellitus. Regarding tumor characteristics, most cases were grade 1 or 2 (35.9% and 41.6%, respectively), whereas 22.5% were grade 3. The majority of tumors were diagnosed at FIGO stage IB (49.4%), although a substantial proportion presented with locally advanced or metastatic disease (FIGO III–IV). Lymphovascular space invasion (LVSI) was observed in 17.2% of cases. Lymph node metastasis was confirmed in 52.2% of patients. The median largest tumor dimension was 30.50 (21.50–50.00) mm, with most tumors classified as medium (20–40 mm, 46.7%) or large (>40 mm, 33.3%). The median follow-up time was 31.0 months (IQR: 20.5–51.5).

To further characterize patients according to nodal status, clinicopathological and hematological parameters were compared between patients with and without lymphatic metastasis. As shown in Table 2, patients with lymphatic metastasis were older and showed larger tumor size and higher mean NLR values. PLR and MLR were also slightly higher in the lymph node-positive group. These findings provided the basis for further analyses evaluating the association between inflammatory markers and adverse clinicopathological features.

To visualize the differences in inflammatory markers according to lymphatic metastasis status, NLR, PLR, and MLR values were plotted for patients without and with lymphatic metastasis (Figure 2).

Among the binary clinicopathological variables, NLR was associated with lymph node metastasis (Mann–Whitney U = 746.0; unadjusted *p* = 0.015), whereas PLR and MLR showed no significant associations with nodal status. LVSI was associated with NLR (Mann–Whitney U = 257.0; unadjusted *p* < 0.001) and showed a nominal association with MLR (Mann–Whitney U = 410.5; unadjusted *p* = 0.048), whereas PLR was not significant (unadjusted *p* = 0.101). No significant associations were observed between the evaluated inflammatory markers and p53 status, p16 status, or tumor ulceration. For p53 status, the corresponding unadjusted *p* values were 0.676 for NLR, 0.210 for PLR, and 0.263 for MLR. For p16 status, the unadjusted *p* values were 0.149, 0.494, and 0.108, respectively. For tumor ulceration, the unadjusted *p* values were 0.219, 0.433, and 0.168, respectively. After FDR correction, the associations of NLR with lymph node metastasis (q = 0.047) and LVSI (q = 0.006) remained statistically significant, whereas the nominal association between MLR and LVSI did not remain statistically significant (q = 0.308). To further illustrate the association between inflammatory markers and LVSI, NLR, PLR, and MLR values according to LVSI status are shown in Figure 3.

To summarize the overall association pattern, *p* values for the associations between inflammatory markers and binary clinicopathological variables are shown in Figure 4.

**Table 3:** Correlations between inflammatory markers and continuous clinicopathological variables.

Clinicopathological Variable	Marker	Statistic (ρ)	*p* Value	Significant
Maximum depth of stromal invasion	NLR	0.139	0.208	No
Maximum depth of stromal invasion	PLR	0.025	0.822	No
Maximum depth of stromal invasion	MLR	0.047	0.676	No
Largest tumor dimension	NLR	0.190	0.073	No
Largest tumor dimension	PLR	0.114	0.286	No
Largest tumor dimension	MLR	0.133	0.215	No

Correlations were assessed using Spearman’s rank correlation analysis. Values are presented as Spearman’s correlation coefficients (ρ) with corresponding *p* values. NLR, neutrophil-to-lymphocyte ratio; PLR, platelet-to-lymphocyte ratio; MLR, monocyte-to-lymphocyte ratio.

### 4.1. Diagnostic Performance: Lymphatic Metastasis

**Figure 5:**
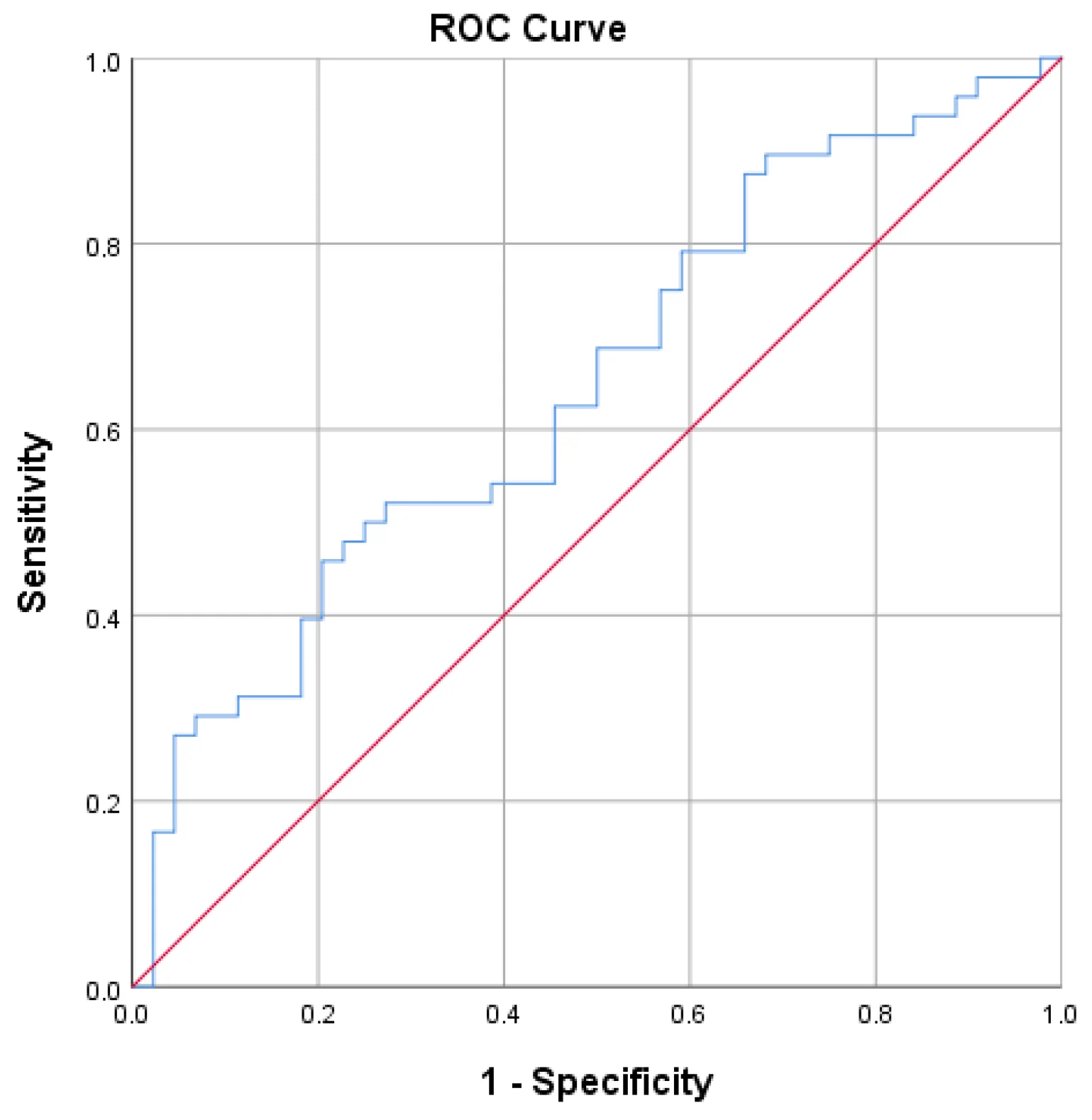
Receiver Operating Characteristic (ROC) curve for preoperative NLR for lymphatic metastasis. The ROC curve demonstrates the discriminatory performance of the neutrophil-to-lymphocyte ratio (NLR) for lymph node metastasis. Sensitivity (true positive rate) is shown on the y-axis and 1-specificity (false positive rate) on the x-axis. The area under the curve (AUC) was 0.647 (95% confidence interval [CI]: 0.535–0.759; unadjusted *p* = 0.015; FDR-adjusted q = 0.047), indicating statistically significant but limited discriminatory performance.

ROC curve analysis was used to assess the discriminatory performance of inflammatory markers for adverse clinicopathological outcomes. For lymph node metastasis, NLR showed modest but statistically significant discriminatory performance (AUC = 0.647; 95% CI: 0.535–0.759; unadjusted *p* = 0.015; FDR-adjusted q = 0.047; Figure 5).

An exploratory ROC-derived NLR cut-off of approximately 3.1 using the Youden index had a sensitivity of 45.8%, a specificity of 79.5%, and an overall accuracy of 62.0% for lymph node metastasis. The Closest Top-Left method yielded a cut-off of approximately 2.83, with a sensitivity of 52.1%, a specificity of 72.7%, and an accuracy of 62.0%. Because both thresholds were derived and evaluated in the same cohort, these estimates represent apparent in-sample performance and may be optimistic. Table 4 summarizes the results.

Using the Youden-based cut-off, patients were grouped according to NLR values below or above 3.1. Lymphatic metastasis was observed in 22 of 31 patients with NLR ≥ 3.1, compared with 26 of 61 patients with lower NLR values. This difference was statistically significant in cross-tabulation analysis (Pearson χ^2^ *p* = 0.010; Fisher’s exact *p* = 0.015). The odds ratio (OR) for lymphatic metastasis in patients with NLR ≥ 3.1 was 3.291 (95% CI: 1.302–8.313). The corresponding distribution is presented in Table 5.

Logistic regression analysis was performed to further evaluate the association between elevated NLR and lymphatic metastasis. In the univariable model, NLR ≥ 3.1 was significantly associated with lymphatic metastasis (OR = 3.291; 95% CI: 1.302–8.313; *p* = 0.012). Multivariable analyses were subsequently performed in the 79 patients with complete data for all included variables.

Model 1 included elevated NLR, largest tumor dimension, tumor grade, maximum depth of stromal invasion, and LVSI. Elevated NLR was not significantly associated with lymph node metastasis (adjusted OR = 1.296; 95% CI: 0.417–4.032; *p* = 0.654). Largest tumor dimension (adjusted OR = 1.020 per mm; 95% CI: 0.990–1.050; *p* = 0.195), tumor grade (adjusted OR = 1.452; 95% CI: 0.726–2.902; *p* = 0.292), and maximum depth of stromal invasion (adjusted OR = 1.067 per mm; 95% CI: 0.968–1.175; *p* = 0.191) were also not statistically significant. LVSI had an adjusted odds ratio of 5.227 for lymph node metastasis; however, the estimate did not reach statistical significance and was highly imprecise, as reflected by the wide confidence interval (95% CI: 0.945–28.905; *p* = 0.058). Model 2 excluded LVSI and included elevated NLR, largest tumor dimension, tumor grade, and maximum depth of stromal invasion. The estimated association for elevated NLR was higher but remained statistically non-significant (adjusted OR = 1.823; 95% CI: 0.640–5.192; *p* = 0.261). Largest tumor dimension (adjusted OR = 1.019 per mm; 95% CI: 0.990–1.048; *p* = 0.201), tumor grade (adjusted OR = 1.349; 95% CI: 0.696–2.613; *p* = 0.375), and maximum depth of stromal invasion (adjusted OR = 1.081 per mm; 95% CI: 0.981–1.193; *p* = 0.117) were not statistically significant.

Both models were statistically significant overall and showed adequate goodness of fit. Model 1 had an omnibus χ^2^ of 17.593 (*p* = 0.004), a Cox–Snell R^2^ of 0.200, a Nagelkerke R^2^ of 0.267, a Hosmer–Lemeshow *p* value of 0.315, and an overall classification accuracy of 67.1%. Model 2 had an omnibus χ^2^ of 13.350 (*p* = 0.010), a Cox–Snell R^2^ of 0.155, a Nagelkerke R^2^ of 0.208, a Hosmer–Lemeshow *p* value of 0.871, and an overall classification accuracy of 69.6%. The results of both multivariable models are summarized in Table 6.

These findings indicate that although elevated NLR was associated with lymphatic metastasis in univariable analysis, this association was not maintained after adjustment for established clinicopathological factors. Therefore, NLR should be interpreted as a marker associated with adverse pathological features rather than as an independent predictor of nodal metastasis.

#### Exploratory Subgroup Analysis in Patients with FIGO Stage IB–II Disease

An exploratory ROC analysis was performed in patients with FIGO stage IB–II disease and evaluable nodal status. The analysis included 47 patients, of whom 5 had lymph node metastasis. NLR did not show statistically significant discriminatory ability for nodal metastasis in this subgroup (AUC = 0.552; 95% CI: 0.273–0.831; *p* = 0.704). The estimate was imprecise because of the limited number of node-positive cases.

### 4.2. Diagnostic Performance: LVSI

**Figure 6:**
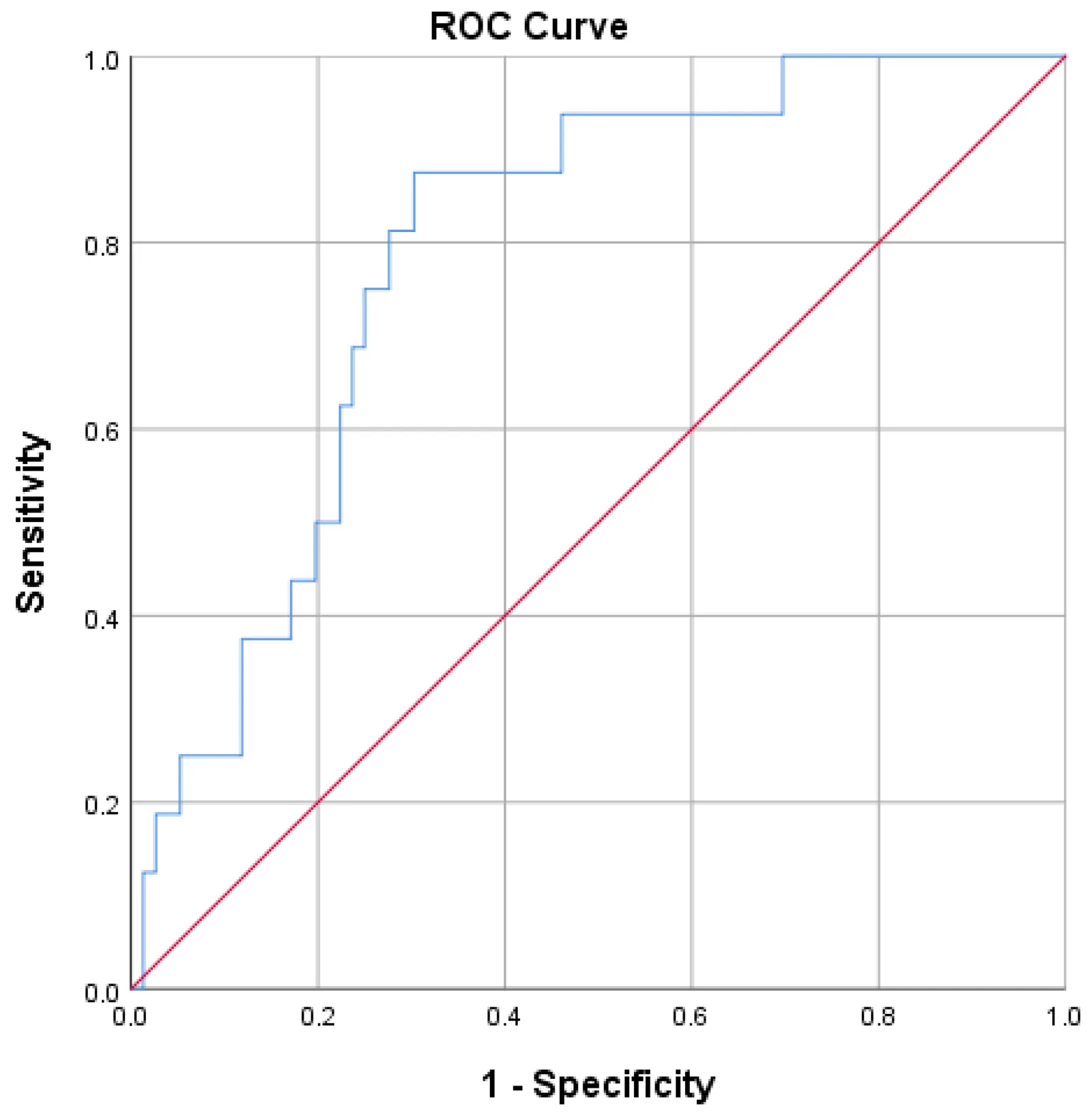
Receiver Operating Characteristic (ROC) curve for preoperative NLR for LVSI. The ROC curve demonstrates the discriminatory performance of the NLR for the presence of LVSI. The AUC was 0.789 (95% CI: 0.683–0.894; unadjusted *p* < 0.001; FDR-adjusted q = 0.006).

ROC curve analysis was also performed to evaluate the discriminatory performance of inflammatory markers for the presence of LVSI (Figure 6). Among the evaluated markers, NLR had the highest AUC for LVSI, with an AUC of 0.789 (95% CI: 0.683–0.894; unadjusted *p* < 0.001; FDR-adjusted q = 0.006). However, this analysis was based on only 16 LVSI-positive cases. At the Youden index-based cut-off, the PPV was 0.378, indicating limited ability to confirm the presence of LVSI among patients classified as high risk. MLR showed a nominal association with LVSI in the unadjusted analysis (AUC = 0.658; 95% CI: 0.521–0.795; unadjusted *p* = 0.048), but this finding did not remain statistically significant after FDR correction (q = 0.308). PLR did not reach statistical significance (AUC = 0.631; 95% CI: 0.481–0.781; unadjusted *p* = 0.101). Using the Youden index method, the optimal NLR cut-off value for LVSI was 2.832, with a sensitivity of 87.5%, specificity of 69.7%, and negative predictive value of 96.4%. The diagnostic performance measures for LVSI are summarized in Table 7.

### 4.3. Diagnostic Performance: Tumor Size

**Figure 7:**
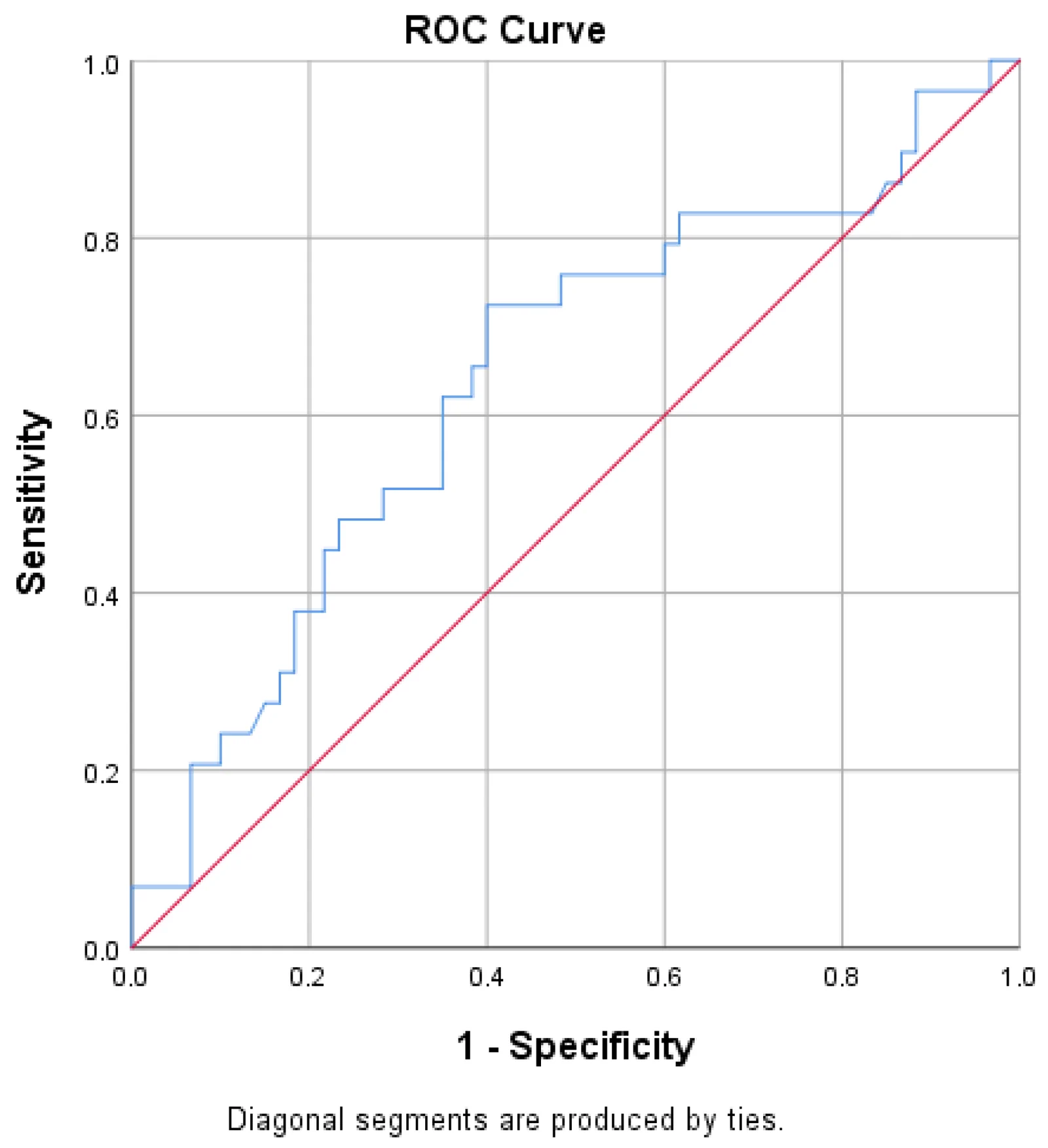
Receiver Operating Characteristic (ROC) curve for preoperative MLR for large tumor size. The ROC curve demonstrates the discriminatory performance of the monocyte-to-lymphocyte ratio (MLR) for large tumor size, defined as a largest tumor dimension >40 mm. The AUC was 0.643 (95% CI: 0.517–0.768; unadjusted *p* = 0.030), indicating modest discrimination in the unadjusted analysis; however, the finding did not remain statistically significant after FDR correction (q = 0.308).

ROC curve analysis was further performed to evaluate the discriminatory performance of inflammatory markers for large tumor size, defined as a largest tumor dimension > 40 mm (Figure 7). NLR showed modest, non-significant discrimination (AUC = 0.618; 95% CI: 0.493–0.743; unadjusted *p* = 0.068). MLR showed modest discrimination in the unadjusted analysis (AUC = 0.643; 95% CI: 0.517–0.768; unadjusted *p* = 0.030), but the finding did not remain statistically significant after FDR correction (q = 0.308). PLR did not show statistically significant discrimination (AUC = 0.535; 95% CI: 0.405–0.664; unadjusted *p* = 0.593). Using the Youden index method, the exploratory MLR cut-off value was 0.304, with a sensitivity of 72.4%, specificity of 60.0%, and negative predictive value of 81.8%. The diagnostic performance measures for large tumor size are summarized in Table 8.

## 5. Discussion

Blood count-derived inflammatory markers, including NLR, PLR, and MLR, have been extensively investigated in several malignancies; however, evidence regarding their associations with clinicopathological characteristics in vulvar squamous cell carcinoma remains limited. The present study aimed to provide a disease-specific evaluation of these inexpensive and routinely available inflammatory indices in relation to lymph node metastasis, LVSI, and tumor size in patients with VSCC. Because LVSI and largest tumor dimension were obtained from definitive pathological assessment, their associations with preoperative inflammatory markers should be interpreted as clinicopathological associations rather than as evidence of validated preoperative predictive utility. In our cohort, preoperative NLR was associated with lymph node metastasis and LVSI.

Systemic inflammation has been increasingly recognized as an important component of cancer progression, influencing tumor growth, invasion, angiogenesis, and metastatic dissemination [6]. Among complete blood count-derived inflammatory markers, NLR has gained particular attention because it is one of the most extensively studied inflammatory indices and has been associated with cancer outcomes across several malignancies [25]. However, data specifically addressing the association between pretreatment inflammatory markers and nodal involvement in vulvar cancer remain limited.

A biologically reasonable explanation for these findings is that elevated NLR may reflect both increased neutrophil-driven tumor-promoting inflammation and a relative reduction in lymphocyte-mediated antitumor immune activity. Neutrophils can contribute to tumor progression through several mechanisms, including the release of pro-angiogenic mediators such as vascular endothelial growth factor (VEGF), extracellular matrix remodeling, and the formation of neutrophil extracellular traps, which may support tumor cell invasion, immune evasion, lymphovascular invasion, and metastatic dissemination [7]. These mechanisms may help explain why higher NLR values were associated with LVSI and nodal involvement in our cohort. However, because the association between elevated NLR and lymphatic metastasis did not remain statistically significant in either multivariable model, NLR should be interpreted as a marker reflecting an adverse tumor–host inflammatory state rather than as an independent determinant of nodal spread or direct evidence of a causal mechanism. In a study of 64 patients with vulvar squamous cell carcinoma, Ertas et al. reported lymph node involvement in 19 cases (29.7%) and found that both preoperative NLR and PLR were significantly higher in node-positive patients. In that study, ROC curve analysis was used to identify the best NLR and PLR values for predicting lymph node metastasis and reported an optimal NLR cut-off value of 2.81, with a sensitivity of 84.5% and specificity of 89.5%; however, the exact cut-off selection criterion was not specified [26]. Similarly, Winarto et al. evaluated 86 patients with vulvar cancer and found that NLR ≥ 2.83 was independently associated with lymph node metastasis, with an adjusted odds ratio of 4.15 (*p* = 0.014). In contrast to Ertas et al., Winarto et al. determined optimal cut-off values using ROC curve analysis based on the highest Youden index [27]. In our cohort, the optimal NLR cut-off values were within a comparable range, with a Youden-based cut-off of 3.1 and a Closest Top-Left cut-off of 2.832. Therefore, although the reported NLR thresholds were very similar across studies, a direct comparison of cut-off values should be interpreted with caution because of differences in cut-off selection methods, patient populations and sample size. Elevated NLR was significantly associated with lymph node metastasis in univariable analysis. However, this association did not remain statistically significant in either multivariable model. Elevated NLR was not independently associated with nodal metastasis after adjustment for largest tumor dimension, tumor grade, and maximum depth of stromal invasion, either with or without LVSI. Consequently, NLR should be interpreted as an inflammatory marker associated with adverse pathological characteristics rather than as an independent predictor of nodal involvement. In the exploratory analysis restricted to patients with FIGO stage IB–II disease, the discriminatory ability of NLR for nodal metastasis was not confirmed. However, this estimate was imprecise because only five patients in the subgroup had lymph node metastasis. The multivariable regression results should also be interpreted cautiously because the relatively limited complete-case sample and the low number of LVSI-positive cases resulted in wide confidence intervals and reduced precision of the adjusted estimates. Consequently, these analyses should be regarded as exploratory and require confirmation in larger prospective cohorts.

Although several ROC analyses reached statistical significance, the observed AUC values for lymph node metastasis and tumor size were only modest. These findings indicate that inflammatory markers alone have limited discriminatory ability and are unlikely to be clinically useful as standalone predictive tools.

Beyond nodal involvement, NLR was also significantly associated with LVSI in our cohort. For NLR, the highest AUC among the investigated outcomes was observed for LVSI. However, this estimate was based on only 16 LVSI-positive cases, and the low PPV limits its clinical interpretability. These findings may suggest a relationship between systemic inflammatory status and lymphovascular tumor characteristics, but they should be interpreted cautiously. Although LVSI is only determined after definitive pathological examination and therefore cannot directly influence preoperative treatment decisions, it represents one of the most important pathological indicators of aggressive tumor behavior. Consequently, the observed association between elevated preoperative NLR and LVSI should not be interpreted as prediction of a postoperative finding per se, but rather as evidence that systemic inflammatory status may reflect the biological aggressiveness of the underlying tumor before surgery. Direct evidence regarding the association between blood count-derived inflammatory markers and LVSI or tumor size in VSCC remains limited. Therefore, findings from other gynecologic malignancies may provide useful context, although they should be interpreted with caution. In a cohort of 763 patients with endometrial carcinoma, Temur et al. reported that higher preoperative NLR and PLR values were associated with LVSI and other adverse pathological features. Similarly, Ronsini et al. found that inflammatory indices, including NLR, MLR, and PLR, were significantly related to LVSI positivity in stage I endometrial carcinoma [16,28]. These findings support our observation that NLR may be linked to lymphovascular tumor characteristics, although direct evidence in VSCC remains limited.

The relationship between inflammatory markers and tumor size was less consistent. MLR showed modest discrimination for large tumor size in the unadjusted analysis, but the association did not remain statistically significant after FDR correction. Direct evidence regarding the association between blood count-derived inflammatory markers and tumor size in VSCC remains limited, and comparisons with other gynecologic malignancies should therefore be interpreted with caution. Previous studies have reported inconsistent findings: Li et al. found positive correlations between tumor size and PLR, NLR, and MLR in stage IIB cervical cancer [24], whereas Muangto et al. found that neither NLR nor PLR significantly predicted tumor size ≥ 20 mm in endometrial cancer [19]. In contrast to studies restricted to a specific disease stage or different tumor types, our study evaluated a VSCC cohort that included patients across the full FIGO-stage spectrum. These differences may reflect variations in tumor biology, disease stage, sample size, and cut-off definitions across studies.

In contrast to NLR, PLR and MLR showed less consistent associations with the investigated clinicopathological parameters. Ertas et al. reported higher PLR values in node-positive VSCC patients and identified a PLR cut-off value of 139.5 for predicting lymph node metastasis, with a sensitivity of 68.9% and specificity of 89.5%; however, in our cohort, PLR was not significantly associated with lymphatic metastasis, LVSI, or large tumor size [26]. Evidence specifically regarding MLR in vulvar cancer remains very limited, and its independent prognostic role, particularly for nodal involvement, has not been clearly established. In our cohort, MLR showed nominal associations with LVSI and large tumor size, but neither remained statistically significant after FDR correction, and MLR was not associated with lymphatic metastasis. Overall, NLR showed the most consistent association pattern among the evaluated indices, although its clinical discriminatory performance remained limited. Although NLR showed statistically significant discriminatory ability for lymphatic metastasis, its overall diagnostic performance was modest, with an AUC of 0.647. The exploratory Youden-derived cut-off of approximately 3.1 provided higher specificity than sensitivity, but its overall classification performance remained limited. Because the cut-off values were both derived and evaluated in the same dataset, the reported diagnostic performance represents apparent rather than validated performance and may therefore be optimistic. Independent validation in external cohorts is required before these thresholds can be considered for clinical use. Taken together, these findings support interpreting NLR as an inflammatory marker associated with an adverse pathological profile rather than as a standalone diagnostic or predictive tool. Vulvar squamous cell carcinoma is a rare malignancy, and the development of new therapeutic approaches is challenging because of the limited number of patients available for clinical investigation. In this context, emerging immunotherapeutic strategies, including nanotechnology-based cancer vaccines, may represent potential future treatment options by improving tumor antigen delivery and antitumor immune activation. However, their application in VSCC remains entirely investigational and will require dedicated disease-specific preclinical and clinical studies before any potential clinical role can be established [29,30,31].

Although NLR was associated with adverse pathological characteristics, the present study was not designed to establish its incremental value beyond existing preoperative clinical assessment or imaging. Consequently, the current findings should not be interpreted as supporting the clinical implementation of NLR as a standalone decision-making tool. Rather, they provide evidence that systemic inflammatory status may reflect more aggressive tumor biology in VSCC and warrant further evaluation in larger prospective multicenter cohorts incorporating formal prediction model comparisons.

### 5.1. Limitations

This study has several limitations. Its retrospective, single-center design should be considered when interpreting the findings. The exclusion of patients with unavailable preoperative hematological data or incomplete pathological documentation may have introduced selection bias and limited the generalizability of the findings. In addition, because vulvar squamous cell carcinoma is a rare malignancy, the number of eligible patients was relatively limited. The relatively small sample size may have reduced the ability to detect weaker associations, especially for PLR and MLR. The LVSI analysis was based on only 16 positive cases, and the low PPV limits the clinical interpretation of the corresponding cut-off. The limited complete-case sample and the small number of LVSI-positive patients also reduced the stability and precision of the multivariable estimates, as reflected by the wide confidence interval observed for LVSI. Inflammatory markers may also be influenced by conditions unrelated to the underlying malignancy. Information on potential confounders, including acute or subclinical infection, smoking status, corticosteroid use, and chronic inflammatory conditions, was not systematically available and therefore could not be included in the adjusted analyses. The cut-off values were derived and evaluated within the same cohort without internal or external validation; consequently, the reported diagnostic performance represents apparent rather than validated performance and may be optimistic. Although FDR correction was applied, the large number of exploratory comparisons and the post hoc subgroup analysis increase the risk of unstable findings. Finally, although follow-up data were available, the present study focused on pathological endpoints at primary surgery and did not evaluate recurrence or survival outcomes. Future studies should determine whether the observed associations translate into clinically meaningful differences in long-term oncological outcomes. Further validation in larger, multicenter cohorts is required.

### 5.2. Implications for Practice

Preoperative NLR was associated with adverse pathological characteristics in VSCC. Because NLR can be readily calculated from routine complete blood count results, it represents a simple and widely available inflammatory marker. However, its discriminatory performance for lymph node metastasis was modest, and it was not independently associated with nodal involvement in either multivariable model. Its incremental value beyond standard clinicopathological or imaging-based assessment was not evaluated. Therefore, NLR should be interpreted cautiously and should not be used as a standalone clinical decision-making tool.

## 6. Conclusions

Overall, preoperative NLR was associated with lymphatic metastasis and LVSI in patients with VSCC. However, elevated NLR was not independently associated with nodal metastasis after adjustment for largest tumor dimension, tumor grade, and maximum depth of stromal invasion, either with or without LVSI. These findings suggest that NLR reflects an adverse pathological profile rather than acting as an independent predictor of nodal involvement. Its modest discriminatory performance does not support standalone clinical use. Nominal findings involving MLR did not remain statistically significant after correction for multiple testing, while PLR showed no consistent associations. Further validation in larger independent multicenter cohorts is required.

## Figures and Tables

**Table 1:** Baseline characteristics of the included patients.

Variable	Value
No. of patients	93
Mean age	68.43 ± 11.22
Median age	70
Mean BMI	28.14 ± 5.46
Median BMI	27.85
Hypertension	64/93 (68.8%)
Diabetes mellitus	21/93 (22.6%)
Tumor grade	
Grade 1	32/89 (35.9%)
Grade 2	37/89 (41.6%)
Grade 3	20/89 (22.5%)
Tumor stage	
FIGO IA	2 (2.2%)
FIGO IB	46 (49.4%)
FIGO II	2 (2.2%)
FIGO IIIA	14 (15.0%)
FIGO IIIB	10 (10.7%)
FIGO IIIC	15 (16.1%)
FIGO IVA	2 (2.2%)
FIGO IVB	2 (2.2%)
Lymphatic metastasis (yes)	48/92 (52.2%) ^1^
p53 aberrant	32/53 (60.4%)
p16 positive	20/61 (32.8%)
Ulceration present	46/93 (49.5%)
LVSI present	16/93 (17.2%)
Tumor size (<20 mm)	18/90 (20.0%)
Tumor size (20–40 mm)	42/90 (46.7%)
Tumor size (>40 mm)	30/90 (33.3%)
Maximum depth of stromal invasion (mm)	7.00 (3.00–11.00) ^2^
Largest tumor dimension (mm)	30.50 (21.50–50.00) ^2^
Follow-up time (months)	31.00 (20.50–51.50) ^2^
Calculated follow-up time (months)	20.40 (8.97–34.21) ^2^
Calculated follow-up time (days)	621 (273–1041.5) ^2^

Values are presented as mean ± standard deviation, *n* (%), or median (interquartile range), as appropriate. ^1^ Nodal status was evaluable in 92 patients; one patient underwent local excision without nodal assessment. ^2^ Values are presented as median (interquartile range). Percentages were calculated using the number of patients with available data for each variable. For tumor grade and FIGO stage, percentages were reported to one decimal place using a total-preserving rounding procedure; the underlying counts and denominators were unchanged. Missing data were present for tumor grade (*n* = 4) and largest tumor dimension (*n* = 3). p53 and p16 status were not available for all patients because these markers were not routinely assessed in earlier pathological examinations during the study period. BMI, body mass index; FIGO, International Federation of Gynecology and Obstetrics; LVSI, lymphovascular space invasion.

**Table 2:** Comparison of clinicopathological and hematological characteristics according to lymphatic metastasis status.

Characteristics	Lymph Node Negative (*n* = 44)	Lymph Node Positive (*n* = 48)
Age (years)	67.02 ± 10.67	70.10 ± 11.42
Histopathological grade		
I	18 (40.9%)	14 (29.2%)
II	16 (36.4%)	21 (43.7%)
III	8 (18.2%)	11 (22.9%)
Unknown	2 (4.5%)	2 (4.2%)
Tumor size (mm)	30.60 ± 17.40	47.70 ± 26.50
Neutrophil count ^1^	4.87 ± 2.95	5.84 ± 2.38
Lymphocyte count ^1^	2.15 ± 1.30	1.99 ± 0.90
Monocyte count ^1^	0.61 ± 0.29	0.61 ± 0.20
Platelet count ^1^	0.27 ± 0.07	0.29 ± 0.10
NLR ^2^	2.56 ± 1.50	3.35 ± 1.75
PLR ^3^	142.20 ± 51.80	165.20 ± 73.30
MLR ^4^	0.30 ± 0.10	0.34 ± 0.12

Values are expressed as mean ± standard deviation or *n* (%), as appropriate. ^1^ Neutrophil, lymphocyte, and monocyte counts are presented in G/L, whereas platelet counts are presented in T/L. For PLR calculation, platelet counts were converted to G/L before division by the lymphocyte count. NLR, PLR, and MLR are dimensionless ratios.^2^ NLR, neutrophil-to-lymphocyte ratio; ^3^ PLR, platelet-to-lymphocyte ratio; ^4^ MLR, monocyte-to-lymphocyte ratio.

**Table 4:** Exploratory ROC-derived NLR cut-off values for lymph node metastasis determined using the Youden index and Closest Top-Left methods.

Method	Cut-Off	Sensitivity	1-Specificity	PPV	NPV	+LR	−LR	Accuracy
Youden index method	3.1	0.458	0.205	0.710	0.574	2.24	0.68	0.620
Closest Top-Left method	2.83	0.521	0.273	0.676	0.582	1.91	0.66	0.620

Sensitivity, 1-specificity, PPV, NPV, +LR, −LR, and overall accuracy are shown for each cut-off. NLR, neutrophil-to-lymphocyte ratio; PPV, positive predictive value; NPV, negative predictive value; +LR, positive likelihood ratio; −LR, negative likelihood ratio. The thresholds and corresponding performance estimates were derived and evaluated in the same cohort and were not internally or externally validated; they therefore represent apparent in-sample performance.

**Table 5:** Distribution of lymphatic metastasis according to the Youden-based NLR cut-off.

Lymphatic Metastasis	Low NLR < 3.1	High NLR ≥ 3.1	Total
No	35	9	44
Yes	26	22	48
Total	61	31	92 *

An NLR value ≥ 3.1 was associated with increased odds of lymphatic metastasis (OR = 3.291; 95% CI: 1.302–8.313). * Nodal status was evaluable in 92 patients; one patient underwent local excision without nodal assessment. The Youden-derived threshold of 3.1 was used consistently in the categorical and regression analyses and should be considered exploratory rather than a validated clinical cut-off. OR, odds ratio.

**Table 6:** Multivariable logistic regression analyses of factors associated with lymph node metastasis.

Variable	Model 1			Model 2		
	Adjusted OR	95% CI	*p* Value	Adjusted OR	95% CI	*p* Value
NLR high (≥3.1)	1.296	0.417–4.032	0.654	1.823	0.640–5.192	0.261
Largest tumor dimension (per mm increase)	1.020	0.990–1.050	0.195	1.019	0.990–1.048	0.201
Tumor grade	1.452	0.726–2.902	0.292	1.349	0.696–2.613	0.375
Maximum depth of stromal invasion (per mm increase)	1.067	0.968–1.175	0.191	1.081	0.981–1.193	0.117
LVSI present	5.227	0.945–28.905	0.058	—	—	—

Model 1 included elevated NLR, largest tumor dimension, tumor grade, maximum depth of stromal invasion, and LVSI. Model 2 included elevated NLR, largest tumor dimension, tumor grade, and maximum depth of stromal invasion without LVSI. Complete-case analysis was performed (*n* = 79). Elevated NLR was defined as NLR ≥ 3.1. OR, odds ratio; CI, confidence interval; LVSI, lymphovascular space invasion; NLR, neutrophil-to-lymphocyte ratio.

**Table 7:** Youden index-based diagnostic performance of NLR for LVSI.

Diagnostic Measure	Value
Outcome	LVSI
Marker	NLR
AUC (95% CI)	0.789 (0.683–0.894)
Unadjusted *p* value	<0.001
FDR-adjusted q value	0.006
Method	Youden index
Cut-off	2.832
Sensitivity	0.875
1-Specificity	0.303
PPV	0.378
NPV	0.964
+LR	2.89
−LR	0.18
Accuracy	0.728

The table presents the AUC, unadjusted *p* value, FDR-adjusted q value, optimal cut-off, sensitivity, 1-specificity, PPV, NPV, +LR, −LR, and overall accuracy. AUC, area under the curve; CI, confidence interval; PPV, positive predictive value; NPV, negative predictive value; +LR, positive likelihood ratio; −LR, negative likelihood ratio; NLR, neutrophil-to-lymphocyte ratio; LVSI, lymphovascular space invasion.

**Table 8:** Youden index-based diagnostic performance of inflammatory markers for large tumor size (>40 mm).

Marker	AUC (95% CI)	Unadjusted *p* Value	Cut-Off	Sensitivity	1-Specificity	PPV	NPV	+LR	−LR	Accuracy
NLR	0.618 (0.493–0.743)	0.068	2.422	0.667	0.433	0.435	0.773	1.54	0.59	0.600
PLR	0.535 (0.405–0.664)	0.593	159.197	0.433	0.300	0.419	0.712	1.44	0.81	0.611
MLR	0.643 (0.517–0.768)	0.030	0.304	0.724	0.400	0.467	0.818	1.81	0.46	0.640

The table presents the AUC, unadjusted *p* value, optimal cut-off, sensitivity, 1-specificity, PPV, NPV, +LR, −LR, and overall accuracy for each marker. Displayed *p* values are unadjusted; FDR-adjusted results are reported in the text. AUC, area under the curve; CI, confidence interval; PPV, positive predictive value; NPV, negative predictive value; +LR, positive likelihood ratio; −LR, negative likelihood ratio; NLR, neutrophil-to-lymphocyte ratio; PLR, platelet-to-lymphocyte ratio; MLR, monocyte-to-lymphocyte ratio.

## Data Availability

The data supporting the findings of this study can be obtained by contacting the corresponding author upon request.

## Abbreviations

The following abbreviations are used in this manuscript:

VSCC	vulvar squamous cell carcinoma
NLR	neutrophil-to-lymphocyte ratio
PLR	platelet-to-lymphocyte ratio
MLR	monocyte-to-lymphocyte ratio
LVSI	lymphovascular space invasion
ROC	receiver operating characteristic
AUC	area under the curve
CI	confidence interval
PPV	positive predictive value
NPV	negative predictive value
+LR	positive likelihood ratio
−LR	negative likelihood ratio
BMI	body mass index
FDR	false discovery rate
FIGO	International Federation of Gynecology and Obstetrics
HPV	human papillomavirus
IQR	interquartile range
OR	odds ratio
VEGF	vascular endothelial growth factor
